# Evaluation of Proximate Composition, Physicochemical Properties, and Sensory Attributes of Instant Flour from Brewery Spent Grain, by Blending with Maize (*Zea mays* L.) and Germinated Chickpea (*Cicer arietinum* L.)

**DOI:** 10.1155/2024/2352758

**Published:** 2024-05-31

**Authors:** Wosen Ewketu Mekonnen, Engeda Dessalegn Augchew, Zemenu Kerie Terefe

**Affiliations:** ^1^ BGI Ethiopia St. George Brewery, Addis Ababa, Ethiopia; ^2^ Department of Chemistry Hawassa College of Teacher Education, Hawassa, Ethiopia; ^3^ School of Nutrition Food Science and Technology Hawassa University, Hawassa, Ethiopia

## Abstract

Brewer's spent grain (BSG) is a nutritional-rich by-product of the brewing industry used in different food product development processes. “Corn Soya Blend” (CSB) is prepared from heat-treated maize and soybeans according to the specifications set by the World Food Program (WFP). Three instant formulations—IF20 (70% maize, 10% chickpea, and 20% BSG), IF15 (70% maize, 15% chickpea, and 15% BSG), and IF10 (70% maize, 20% chickpea, and 10% BSG)—were developed in this research. Proximate composition, functional properties, and antinutritional factors were analysed. The sensory quality of porridge samples developed from the instant flour was evaluated using a consumer-oriented panel (food science and technology students) at a five-point hedonic scale. Accordingly, moisture, crude fibre, crude protein, total ash, and crude fat contents increased significantly (*p* < 0.05) as a result of BSG ratio inclusion. Bulk density decreased significantly (*p* < 0.05) while the BSG proportion increased but water absorption capacity increased when the proportion of BSG increased. Phytate and tannin contents were also increased while the BSG proportion increased. However, an increase in germinated chickpea proportion significantly (*p* < 0.05) decreased phytate and tannin contents. While BSG increased, the overall acceptability of porridge samples decreased, with the exception of mouthfeel. According to this study, up to 15% of BSG, 70% maize, and 15% chickpea could be used for instant flour preparation which has a comparable sensory characteristic with the commercial CSB. Hence, it can be used as a substitute for corn-soya mix.

## 1. Introduction

By-products released from food and beverage industries usually contain highly concentrated nutrients including carbohydrates, proteins, essential amino acids, and appreciable levels of minerals, polyphenols, and lipids which are suitable for microbes' growth [1–3]. If these food by-products are not immediately disposed or utilized to other purposes, insects, rodents, spoilage organisms, or even pathogenic microorganisms could be attracted [4].

Spent grain is produced at the end of every mash during brewing [5]. Its use as a foodstuff, especially for ruminant animals, is immense, however, the large-scale brewery production systems and the nature of immediate decomposition, urging the removal of spent grain from the processing lines and production area [6, 7]. Otherwise, the handling equipment and the production area may not be cleaned and hygienic, which could initiate the growth of spoilage and other organisms and, as a result, affect the whole production. Brewer's spent grain (BSG) constitutes about 85% of the total by-products of the brewing industry [8]. Hence, the disposal of this huge amount of by-product is the bottleneck of the brewing industries [3, 7, 9]. As a result, brewing industries existing in developing countries usually use dumping as a major means of their disposal system for their by-products. This leads to the pollution of the surrounding environments where the by-products disposal process is not the best way to sustain the waste management system of the brewery industry, as the production of different food and nonfood products at a large scale in an environmentally friendly manner is the cross-cutting issue of the existence of the industry in both the developed and developing countries [10].

The current huge amount of BSG produced from the breweries in Ethiopia and their future expansion in these plants is a threat of the existence of the companies in a time where environmental pollution is a cross-cutting issue for the government and the industries, the surrounding environment, and the societies living where the industries exist. Unless the existing and future BSG produced or to be produced in Ethiopia brewing industries will be properly managed and handled or alternative income generation means are established, the production or environmental management cost and/or the devastating effect on the surrounding environment and the societies living in the industries will suffer with the risks related in a sustainable way.

**Figure 1:**
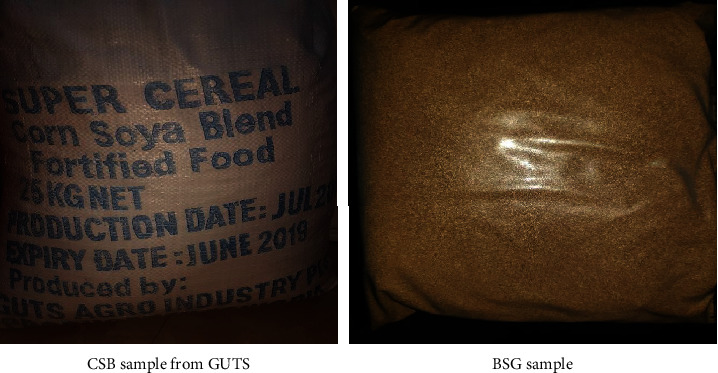
Corn soya blends and brewer's spent grain samples.

On the other hand, the prevalence of malnutrition in Ethiopia is unacceptably high, and as a result, the government of Ethiopia and the collaborating humanitarian institutions have set activity plans on the SEKOTA declaration and invest huge amounts of money to eliminate all forms of malnutrition and zero stunting by 2030. A locally available food-based approach is one of the suggested methods for reducing malnutrition in developing nations, including Ethiopia. It is economical, takes into account locally produced foods, and is technically feasible and socially acceptable to produce on a small or large scale. At the moment, the WFP of the United Nations produces fast foods like CSB, an acronym for maize-soya mix, while Guts Agro Industry and Faffa Food Share Company in Ethiopia mostly distribute them to people in emergency nutrition programs (Figure 1). However, the production of soybean in Ethiopia was estimated to be 66,885 tons in 2012, despite the annual demand being estimated to be 68, 900 tons [11]; excluding export and household processing, the dramatic market demand for soybean is increasing related with lots of poultry farmers that are coming up. To date, limited effort has been made to utilize the BSG coming out of the breweries except for wide use of it in the formulation of animal feeds. However, with hygienic handling and processing, it could be a potential ingredient in the production of blended instant foods, a way of which breweries can turn by-products to wealth. This requires a comprehensive analysis of the nutritive status of the BSG right at production and an evaluation of its suitability for developing an instant food by blending it with locally available staple crops. Studies in other countries have shown that different foods (cakes, bread, biscuits, snacks, and pancakes) can be prepared from BSG by mixing with other ingredients [8, 9, 12, 13] which can be used by children and adults.

Functional properties are the fundamental physicochemical properties that reflect the complex interaction between the composition, structure, and molecular conformation of food components together with the nature of environment in which these are associated and measured [14, 15]. Bulk density is a functional property, defined as the mass of the particles of flour divided by the total volume they occupy [16]. It is an indicator for measuring the quality attributes of food products and has a significant impact on the choice of the product by the consumer. Flours with high bulk density are suitable for applications of food preparation. On the other hand, low bulk density would be useful in the preparation of complementary foods due to their higher volume at constant mass because of higher porosity [17, 18]. Water absorption capacity is the amount of water or moisture absorbed by food or flour to achieve the desirable consistency and create a quality food product [16]. It is important in food application because it is related to mouthfeel properties, thickness, and viscosity [19].

The formulation of nutrient-rich instant precooked food can be used by different age groups. The product can be used in different forms, prepared, and consumed in the form of porridge. In this regard, the output from this study will add a variety to the menu of instant food packed with essential nutrients. One of the objectives of the National Nutrition Program (NNP) is to improve the consumption and utilization of a diversified and nutritious diet in Ethiopia that ensures citizens' optimal health throughout their lives. Therefore, this research contributes to the development of food from locally available ingredients in the country. The developed product can also be an alternative to CSB by mixing BSG with germinated chickpea seeds and maize grain. The utilization of BSG in food product development is an impetus as the strategic means of minimizing the ill effects of mushrooming breweries on the fragile environment too. The use of BSG in the preparation of instant precooked food by blending with chickpea and maize grain is a viable alternative to turning by-products into wealth because this product enhances nutritional values and can be used as a food ingredient for the children and adults. However, to our knowledge, no study has been conducted to use BSG blending with maize and germinated chickpea for the production of instant food. Therefore, the objective of this research was to develop instant maize grain-based food by blending with germinated chickpea seeds and BSG and to evaluate the proximate compositions, physicochemical properties, and sensory attributes of the porridge samples.

## 2. Materials and Methods

### 2.1. Sample Collection and Preparation

In this study, instant precooked food products were developed from locally available food items. For this, widely produced and consumed *Desi*-type chickpea seeds and maize grain were collected from the Hawassa open market while the Brewer's spent grain (BSG) was obtained from Hawassa St. George Brewery factory (BGI) of Ethiopia, and Corn soya blend (CSB) was collected from Guts Agro-Industry P.L.C as a control sample for comparison. BSG flour was prepared according to the method described by Niemi [20]. First, BSG was dried in a hot air oven at 60°C until the moisture reached between 6 and 7%. Then, the dried BSG was ground using a laboratory-grade miller and passed through the sieving size of 0.2 mm. Then, the BSG flour was passed once again to a sieve with a mesh size of 1 mm to remove the remaining husk and packed in a polyethylene bag (Figure 1). Finally, the sample was placed at 4°C in a dry area until the flour was needed for the preparation of instant food composite flour [21]. The chickpea seeds were first cleaned to remove the dust materials and hand sorted also to remove the stones, stalks, and broken undersized and immature grains before winnowing. The germinated chickpea grain was steeped, 1 : 3 w/v, for 24 hours with distilled water and subjected to germination for 24 hours at room temperature. After these treatments, all the germinated chickpea grain was oven-dried until 10-12% moisture was maintained, followed by roasting at 170 to 180°C for 15 minutes by controlling the temperature with an infrared thermometer. Finally, the dried germinated chickpea flour was ground using the laboratory-grade miller (Thomas Wiley laboratory mill, Model 4), and the flour was passed through the sieve with the mesh size of 1 mm to get the final germinated chickpea flour [21]. The maize grain was cleaned and hand sorted to remove stones, dust materials, stalks, and broken undersized and immature grains and winnowed. The maize grain was then subjected to roasting at 145–150°C for 15 minutes using an oven (LTO-FB series) by controlling the temperature with an infrared thermometer. Then, the dried maize grain was ground by a laboratory-grade miller and sieved by using the mesh size of 1 mm to produce the maize flour intended for this study [22].

### 2.2. Formulation of Instant Food Product

Two hundred grams of instant food composite flours was prepared by blending maize, germinated chickpea, and BSG flours with mixing proportions of 70 : 20 : 10, 70 : 15 : 15, and 70 : 10 : 20, considering the WFP CSB (Table 1) recipe formulation and list of compulsory test requirements.

### 2.3. Preparation of Porridge

The porridge samples were prepared by mixing 100 g of each instant four, 4 g of cooking oil, and 1 g of table salt with 235 mL of hot water. The mixture was cooked at 90°C by stirring for 10 min. Then, the product was cooled for further analysis of sensory acceptability.

### 2.4. Experimental Design

The experiment was laid out in a completely randomized design (CRD) with triplicate. The blending combinations consist of three instant foods (IF)—70 : 20 : 10, 70 : 15 : 15, and 70 : 10 : 20—and standard control (Figure 1, WFP CSB formula). The data collected for proximate, antinutritional, and functional properties as well as sensory acceptability of porridge samples developed from the instant flours were analysed using analysis of variance (ANOVA) to see the blending effect. Randomized complete block design (RCBD) was used for sensory data to take care of variability originating from the panelists.

### 2.5. Proximate Analysis

The proximate composition (moisture content, crude protein, crude fat, crude fibre, and total ash) of the sample was determined according to AOAC [23] method. The utilizable carbohydrate content was also determined by the difference of the moisture, crude protein, crude fat, and total ash contents from 100, and gross energy was determined by using Atwater's conversion factor (1 gram protein = 4 kcal, 1 gram carbohydrate = 4 kcal, and 1 gram fat = 9 kcal).

### 2.6. Functional Properties

#### 2.6.1. Bulk Density

Bulk density was determined according to Ijarotimi and Keshinro [24]. Five grams of instant flour was put into an empty 25 mL measuring cylinder. The cylinder was trapped continuously until constant volume was obtained. Then, the bulk density (g/mL) was calculated as the weight of instant flour (g) divided by flour volume (mL).

#### 2.6.2. Water Absorption Capacity

Water absorption capacity (WAC) which gives an indication of the amount of water available for gelatinization was determined according to Adem et al. [25] with slight modification. About 2.5 g of each sample was added to 30 mL distilled water in a weighed 50 mL centrifuge tube. The tubes were agitated for about five minutes before being centrifuged at 4000 rpm for twenty minutes. The mixtures were decanted, and the clear supernatant was discarded. Adhering drops of water were carefully siphoned as much as quantitatively possible, and the tubes were reweighed. WAC is expressed as the weight of water bound by g/100 g dry flour.

### 2.7. Antinutritional Factors

#### 2.7.1. Phytate

Phytate was determined according to Terefe et al. [26] with slight modification. About 0.1 g of flour sample was extracted with 10 mL of 2.4% HCl in a reciprocal shaker (Eberbach E5900) for one hour at an ambient temperature and centrifuged at 3000 rpm for thirty minutes. The clear supernatant was used for phytate estimation. Exactly 2 mL of wade reagent, a mixture of 0.03% solution of FeCl_3_.6H_2_O and 0.3% of sulfosalicylic acid in water, was added to 3 mL of the sample solution (supernatant) and vortexed (Maxi Maxi II) for 5 seconds. The absorbance of the sample solutions was measured at 500 nm using a UV-Vis spectrophotometer (JENWEY, 6300, Switzerland). The phytic acid standard curve (*y* = −0.0073*x* + 0.4242) was made, and the result was expressed in terms of milligram of phytic acid equivalent per hundred of the dried sample (mg PAE/100 g).

#### 2.7.2. Total Condensed Tannin

The condensed tannin content was performed according to the method described by Chew et al. [27]. Exactly 0.5 mL undiluted crude extract was first mixed with 3 mL of vanillin reagent (4%, w/v, in absolute methanol), followed by the addition of 1.5 mL of concentrated HCl (37% w/w, *d* = 1.42 g/mL). Mixtures were stored in a dark environment at room temperature for 15 min. Blank was prepared by replacing 0.5 mL of undiluted crude extract with 0.5 mL of deionized water. The absorbance of the mixture was measured at 500 nm against a blank using a UV light spectrometer (JENWEY, 6300, Switzerland). The absorbance of the blank was subtracted from the absorbance of the corresponding vanillin-containing sample. A catechin standard curve was constructed after correcting for blank, and the linear portions of the curve were extrapolated to produce the standard curve (*y* = 0.004*x* + 0.033, *R*^2^ = 0.995), and the results were expressed as milligram of catechin equivalent per 100 g dry weight sample (mg CE/100 g DW).

### 2.8. Sensory Evaluation

The sensory acceptability was determined by preparing porridge samples in the respective blending ratio and comparing them with the control (CSB) porridge. The acceptability attributes of porridge samples—color, flavour, aroma, and texture (mouthfeel)—and overall acceptability were assessed using forty-eight panelists of fourth-year food science and technology students in the College of Agriculture, Hawassa University, with a five-point hedonic scale (where 1 = dislike very much and 5 = like very much). The panelists were provided with a mouth rinse in between each taste.

### 2.9. Statistical Analysis

The data collected for each parameter were analysed using a Statistical Analytical System (SAS version 9.0). The mean comparison test was carried out using Duncan's multiple range test at *p* < 0.05. The results are reported as mean ± standard deviation.

## 3. Results and Discussion

### 3.1. Proximate Composition

The proximate composition of BSG varies due to the type of beer processing, handling, variety of the barley, season of harvesting, hop type, malt used, and mashing process of malt used [28–30]. In the present study (Table 2), the moisture content of the dried BSG was comparable with the findings of Arranz et al. [31] and Niemi [20], but lower than the findings of [32]. The moisture contents of 70% maize, 20% BSG, and 10% germinated chickpea; 70% maize, 15% BSG, and 15% germinated chickpea; and 70% maize, 10% BSG, and 20% germinated chickpea blends were higher than that of CSB (4.78%). This might be because the blends contained higher fibre contents [33]. Even though the moisture contents of all the blends were higher than the CSB, according to the WFP list of compulsory test requirement, all the blends fulfil the requirement which is a maximum of 10.0% [22]. The moisture contents of blends of 70% maize, 20% BSG, and 10% germinated chickpea; 70% maize, 15% BSG, and 15% germinated chickpea; and 70% maize, 10% BSG, and 20% germinated chickpea were not significantly different (*p* > 0.05). Increasing the BSG contents has no significant effect on the moisture content. However, the moisture contents of these products were significantly higher than that of CSB (Table 2). Similarly, increasing the proportion of germinated chickpea has no significant (*p* > 0.05) effect on the moisture contents of all the blends. According to the study conducted by Yitayew et al. [33], the moisture content of bread was significantly affected (*p* < 0.05) by the BSG level, and it was increased as the BSG level increased from 0 to 20%. Similarly, the work of Fărcaș et al. [34] showed that the moisture content of the bread was higher as the BSG level was increased.

The crude fibre contents of the chickpea and maize were lower than the crude fibre content of BSG used in this study. The crude fibre of the BSG was found to be higher than the finding of Ajanakua et al. [7], however lower than the result found by Senthilkumar et al. [35]. This might be due to the mesh size of the sieve used in this study which was different from the two studies mentioned earlier. The crude fibres of 70% maize, 20% BSG, and 10% germinated chickpea; 70% maize, 15% BSG, and 15% germinated chickpea; and 70% maize, 10% BSG, and 20% germinated chickpea blends were 7.10, 5.62, and 5.59%, respectively, while CSB is 3.59% (Table 2). The fibre contents of 70% maize, 15% BSG, and 15% germinated chickpea and 70% maize, 10% BSG, and 20% germinated chickpea blends were not significantly different (*p* > 0.05), but these values were significantly lower (*p* < 0.05) than that of the blend of 70% maize, 20% BSG, and 10% germinated chickpea. This might be because of the presence of a higher amount of fibre in BSG than that of maize and germinated chickpea (Table 2). The crude fibre of all the blends was significantly higher (*p* < 0.05) than that of the CSB, and all the blends exceeded the maximum requirement set on the WFP list of compulsory test requirement (maximum 4.0%) [22]. Similar results were reported on BSG-enriched maize bread and wheat bread sticks [9, 13]. According to these studies, increasing the BSG proportion increased the fibre content of BSG-enriched maize bread and wheat bread sticks. On the other hand, increasing germinated chickpea content from 10 to 15% has no significant (*p* > 0.05) impact on the crude fibre content while the instant flour with 20% germinated chickpea had the lower fibre content (*p* < 0.05) than the other two blends. This could mainly be due to the lower fibre content in chickpea as compared to BSG (Table 2).

The crude fat content of the BSG flour (Table 2) was higher than the chickpea, but lower than maize. This result is comparable with the crude fat content of BSG found in the study conducted by Niemi [20], however lower than in the study conducted by Kanauchi et al. [1]. The reason might be the variety of the barley, season of harvesting, hop type, malt used, and mashing process of malt used [29]. On the contrary, according to the study conducted by Ajanakua et al. [7], the crude fat content of the BSG flour was much lower than that of the present finding (2.79%). The crude fats of 70% maize, 20% BSG, and 10% germinated chickpea; 70% maize, 15% BSG, and 15% germinated chickpea; and 70% maize, 10% BSG, and 20% germinated chickpea blends were not significantly different (*p* > 0.05) though lower than that of CSB (Table 2). Also, increasing the germinated chickpea showed no significant effect (*p* > 0.05) on the total fat content of the three blends. Although the crude fat content of CSB is slightly higher than all the blends, all the blends fulfil the standard set by the WFP list of compulsory test requirements (minimum 6%) [22].

The crude protein content of the BSG (27.45%) in the present study was higher than that of chickpea (24.79%) and maize (10.09%), which is in line with the finding of Celus et al. [36] for BSG (26.7%) but higher than the values of 19.0 to 24.34%, as reported by Senthilkumar et al. [35] and Russ et al. [37]. This makes BSG as a potential source of protein and is suitable for the substitution of CSB for human consumption. The contribution of maize for protein increment to the blends was the lowest as compared to BSG and chickpea. Although the protein content of CSB and the blends are slightly lower than the standard set on the WFP list of compulsory test requirements (minimum 14.0%) [22], the different blends and the CSB were not significantly different (*p* > 0.05).

The total ash content of the BSG flour was similar with some of the BSG collected from different countries [3, 13, 37], however, lower than that of the results reported by Niemi [20] and Senthilkumar et al. [35] and higher than the findings of Kanauchi et al. [1] and Fărcaș et al. [34]. The possible reason for the variation of total ash in BSG could be the malted barley variety difference and the agroecology of the cultivation area of the raw barley [29]. At the same time, the ash content of BSG in this study is also higher than that of chickpea and maize. As shown in Table 2, the BSG level also had a significant effect (*p* < 0.05) on the ash content of the product. It increased from 2.01 to 2.16% as the BSG level was increased from 10 to 20%. However, increasing the level of germinated chickpea decreased the ash content. This might be attributed to the presence of higher amounts of minerals in the BSG. The addition of bran increases the ash content of the bread [3]. The ash content of CSB is found to be higher than the blends in this study, and this might be due to fortification with “Di-calcium Phosphate Anhydrous” and “Potassium chloride.” However, all the blends still comply with the standard set on the WFP list of compulsory test requirement (maximum 4.1%) [22]. Furthermore, the ash contents of 70% maize, 20% BSG, and 10% chickpea and 70% maize, 15% BSG, and 15% chickpea blends were not significantly different (*p* > 0.05), but these values were significantly higher (*p* < 0.05) than the ash content of the blend containing 70% maize, 10% BSG, and 20% chickpea. This is because the ash content of BSG was greater than that of the germinated chickpea (Table 2).

The total carbohydrate content of the BSG flour was found to be 54.14%, which is comparable with the finding in the study conducted by Ajanakua et al. [7], which was 51.38%. However, this result for BSG, in the present study, was lower than that found in the study conducted by Adeniran et al. [38]. This difference might be related with the mash filtration mechanism implemented. The spent grain separation mechanisms that were used in our case are able to extract more liquid wort, which could reduce the remaining carbohydrate from the BSG. On the other way, the total carbohydrate found in the BSG used for this study was lower than the chickpea and maize, used in the same study. The total carbohydrate contents of IF20, IF15, and IF10 blends were found to be 69.26, 69.69, and 70.08%, respectively, while CSB is 70.08% (Table 2). Even if carbohydrate is not included in the WFP list of compulsory test requirements, the carbohydrate contents of all blends were not significantly different from that of CSB (*p* > 0.05).

The total gross energy of BSG is slightly lower than that of both chickpea and maize (Table 2). The total gross energy of BSG under this study was higher than the findings of Fărcaș et al. [34] and Canedo et al. [39], with the values of 335.43 and 349.81 kcal/100 g, respectively. The comparable gross energy finding of BSG with respect to germinated chickpea and maize is mainly due to the fact that, even if BSG is lower in carbohydrate content, the loss of carbohydrate is seemingly compensated by the high protein content. The gross energy of 70% maize, 20% BSG, and 10% germinated chickpea and 70% maize, 15% BSG, and 15% germinated chickpea blends was not significantly different (*p* > 0.05) from each other, but these values were significantly lower than that of CSB (Table 2).

### 3.2. Functional Properties of Ingredients and Instant Flours

The bulk density of the BSG in the present study (Table 3) was lower than that of the maize and the germinated chickpea. Similarly, bulk density increased significantly (*p* < 0.05) when the BSG proportion in the formulation decreased. The bulk density for blends—70% maize, 20% BSG, and 10% chickpea; 70% maize, 15% BSG, and 15% chickpea; and 70% maize, 10% BSG, and 20% germinated chickpea—were 0.439, 0.448, and 0.475 g/mL, respectively. The bulk density of CSB (control) (0.524 g/mL) was found to be the highest among the blends. The water absorption capacity was shown to be related to the total carbohydrate content [13]. BSG showed higher water holding capacity than that of maize and germinated chickpea. This might be because of the presence of a lower amount of total carbohydrate in BSG as compared to maize and germinated chickpea (Table 2). Similarly, the water absorption capacity of the three blends increased significantly (*p* < 0.05) when the BSG proportion increased (Table 3). The water absorption capacity of CSB (control) was found to be the least of all (131.4 g/100 g) compared to that of maize and germinated chickpea.

### 3.3. Antinutritional Contents of Ingredients and Instant Flours

Phytates are the principal storage form of phosphorus and are particularly abundant in cereals and legumes [40]. Phytate reduces the bioavailability of divalent cations such as calcium, magnesium, zinc, and iron [41]. Germination has been an effective treatment to remove antinutritional factors in cereals. These are the mobilizing secondary metabolic compounds which are thought to function as reserve nutrients [40]. The phytate content of the BSG flour in the present study was 195.66 mg/100 g, which was the highest of all (Table 4). This might be because BSG is mainly composed of the husks and outer layers of the grain, which are not solubilized in mashing [20, 34]. Phytate contents in maize and chickpea were 129.85 and 37.62 mg/100 g, respectively. The steeping and germination of the chickpea reduce the phytate significantly [21]. This could be the main reason that reduces the phytate content of the chickpea in this study. Even though phytate is not listed as a compulsory requirement on WFP's standard, the three blends, IF20, IF15, and IF10, were found to be 133.15, 107.99, and 92.96 mg/100 g, respectively. Particularly, the blend of IF10 was found to be less in phytate content than the CSB (100.96 mg/100 g). The achievement of getting less phytate in the germinated chickpea flours apparently helps in the reduction of phytate in chickpea blends. This could be the main reason that the lower phytate content was observed in a sample containing 20% germinated chickpea.

Tannins are polyphenolic antinutritional biomolecules that bind and precipitate proteins in foods. The total condensed tannin contents of BSG, germinated chickpea, and maize in the present study were 178.51, 95.05, and 13.18 mg/100 g, respectively. The highest content on the BSG is due to the husks and outer layers of the grain, which are not solubilized in mashing [20, 34]. Similarly, the condensed tannin content in germinated chickpea was reduced as a result of steeping and germination of chickpea [21]. The total condensed tannin contents of all three blends (Table 4) were found to be higher (*p* < 0.05) than that of CSB, but there was no significant difference (*p* > 0.05) among the three blends.

### 3.4. Sensory Evaluation of Porridge Products

Sensory properties of a product are important quality parameters, which influence the status of the finished product on the market. Results of scientific research have shown that the quality and consumer acceptability of a product can be identified and controlled with the descriptive analysis or by consumer testing to examine if the overall product quality or the selected property of the product was affected or not [42]. As indicated in Table 5, the color of the porridge made from CSB and 70% maize, 10% BSG, and 20% germinated chickpea blend instant flour was preferred than the others. In addition, preference increases while the BSG proportion decreased. The light brown color of the BSG and the darker brown color of the chickpea flours made the blended porridges under investigation darker than the control. But the color preference, flavour, and taste showed a slight increase with an increasing percentage of germinated chickpea. The flavour attribute of 70% maize, 15% BSG, and 15% germinated chickpea; 70% maize, 10% BSG, and 20% germinated chickpea blends; and CSB was not significantly different (*p* > 0.05), which means that the blending ratio had no much influence on the “flavour” of porridge in this study. However, the flavour attribute of 70% maize, 20% BSG, and 10% germinated chickpea blend was the lowest compared (*p* < 0.05) to other blends and the control. The mean values of aroma attribute of 70% maize, 20% BSG, and 10% germinated chickpea; 70% maize, 15% BSG, and 15% germinated chickpea; and 70% maize, 10% BSG, and 20% germinated chickpea blends were 2.94, 3.41, and 3.41, respectively. The control (CSB) sample had the highest aroma (*p* < 0.05) compared to others. This could be because the grain aroma of the BSG flour affects the porridge aroma. The taste mean values of porridges containing 15 and 10% germinated chickpea and CSB were 3.44, 3.47, and 3.69, respectively. The 70% maize, 20% BSG, and 10% germinated chickpea blend were the least preferred sample based on taste criterion. The mean values of mouthfeel properties of 70% maize, 20% BSG, and 10% germinated chickpea; 70% maize, 15% BSG, and 15% geminated chickpea; 70% maize, 10% BSG, 20% germinated chickpea blends; and CSB were 3.09, 3.47, 3.38 and 3.28, respectively. The blend of 70% maize, 15% BSG, and 15% germinated chickpea scored the highest in terms of mouthfeel compared to other blends and the control. Overall acceptability of the blends containing 10 and 15% germinated chickpea and CSB porridges was not significantly different (*p* > 0.05) from each other, and porridge containing 20% BSG scored significantly (*p* < 0.05) the lowest overall acceptability. In general, the sensory evaluation result has shown that the addition of BSG commonly reduces the acceptability of porridge products, as compared to CSB (control). However, based on the hedonic scale used for this specific study, all attributes were scored above the average value. Overall, the attributes were influenced negatively as the proportion of BSG in the blends increased, and this might be due to the impacts of BSG on physical properties such as processing characteristics and the quality of the final products [13].

## 4. Conclusion

This research evaluated the potential of brewer's spent grain in the production of instant flour by blending with maize and germinated chickpea flours. The instant flour developed from the formulation of 70% maize, 20% germinated chickpea, and 10% BSG and 70% maize, 15% germinated chickpea, and 15% BSG was comparable to the CSB in terms of proximate and sensory acceptability. Therefore, these instant blends can be considered as an alternative of CSB. In terms of protein, BSG could be a good source to be used in different blended instant food products to contribute for the effort to reduce the malnutrition issue in the developing countries. In general, because the fibre properties influenced the physical properties and chemical compositions, it is suggested that fibre modification or other mechanisms should be implemented to reduce the fibre content to improve the color, flavour, aroma, and taste of the blends. Moreover, due to its microbial instability and high perishability, BSG should be further studied to incorporate it in traditional Ethiopian foods for the development of different instant food products. Also, further study should be conducted regarding the reduction of antinutritional factors, micronutrient (mineral, vitamins, and phenolic compounds) contents, microbial safety, and biofunctional activities of BSG from Ethiopia.

## Figures and Tables

**Table 1:** Formulation of instant food composite flour with different proportions.

Ingredients	IF10	IF15	IF20	Control
Maize	70	70	70	—
Chickpea	20	15	10	—
BSG	10	15	20	—
CSB	—	—	—	100

IF10 (70% maize, 20% chickpea, and 10% BSG), IF15 (food 70% maize, 15% chickpea, and 15% BSG), IF20 (70% maize, 10% chickpea, and 20% BSG), and CSB (corn soya blend).

**Table 2:** Proximate composition of ingredients, blends, and control instant food flours.

	Moisture (%)	Crude fibre (%)	Crude fat (%)	Crude protein (%)	Total ash (%)	Total carbohydrate (%)	Gross energy (kcal/100 g)
*Ingredients*							
BSG	7.03 ± 0.11	16.26 ± 1.02	7.12 ± 0.18	27.45 ± 0.46	4.26 ± 0.04	54.14 ± 0.49	390.41 ± 1.45
G. chickpea	5.42 ± 0.03	10.23 ± 0.66	5.40 ± 0.15	24.79 ± 0.18	2.45 ± 0.02	61.94 ± 0.08	395.50 ± 0.91
Maize	7.86 ± 0.01	3.01 ± 0.05	7.25 ± 0.09	10.09 ± 0.13	1.61 ± 0.03	73.19 ± 0.01	398.31 ± 0.30

*Blends and control*							
IF20	7.63 ± 0.14^a^	7.10 ± 0.04^a^	7.27 ± 0.48^b^	13.68 ± 0.18^a^	2.16 ± 0.02^b^	69.26 ± 0.18^a^	397.23 ± 2.85^b^
IF15	7.52 ± 0.29^a^	5.62 ± 0.46^b^	7.12 ± 0.18^b^	13.54 ± 0.00^a^	2.13 ± 0.05b	69.69 ± 0.52^a^	396.98 ± 0.47^b^
IF10	7.40 ± 0.01^a^	5.59 ± 0.42^b^	7.04 ± 0.05^b^	13.47 ± 0.23^a^	2.01 ± 0.00^c^	70.08 ± 0.19^a^	397.56 ± 0.32^b^
CSB (control)	4.78 ± 0.11^b^	3.59 ± 0.00^c^	8.26 ± 0.00^a^	13.55 ± 0.07^a^	3.33 ± 0.02^a^	70.08 ± 0.21^a^	408.88 ± 0.54^a^

IF20 (70% maize, 10% germinated chickpea, and 20%BSG), IF15 (70% maize, 15% germinated chickpea, and 15% BSG), IF10 (70% maize, 20% germinated chickpea, and 10% BSG), and CSB (corn soya blend). Values are mean ± standard deviation; the means with the same superscript letters across the column are not significantly different at *p* < 0.05. All values are taken from triplicate measurements.

**Table 3:** The bulk density and water absorption capacity of ingredients and blend instant flour.

	Bulk density (g/mL)	WAC (g/100 g)
*Ingredients*		
BSG	0.352 ± 0.009	332.20 ± 30.26
G. chickpea	0.545 ± 0.014	162.80 ± 1.70
Maize	0.495 ± 0.007	115.20 ± 3.39

*Blends and control*		
IF20	0.439 ± 0.013^c^	155.60 ± 2.83^a^
IF15	0.448 ± 0.004^bc^	147.40 ± 0.85^b^
IF10	0.475 ± 0.000^b^	140.20 ± 1.41^c^
CSB (control)	0.524 ± 0.016^a^	131.40 ± 0.85^d^

IF20 (70% maize, 10% germinated chickpea, and 20% BSG), IF15 (70% maize, 15% germinated chickpea, and 15% BSG), IF10 (70% maize, 20% germinated chickpea, and 10% BSG), and CSB (corn soya blend). Values are mean ± standard deviation; the means with the same superscript letters across the column are not significantly different at *p* < 0.05. All values are taken from triplicate measurements.

**Table 4:** Antinutritional content of ingredients, blends, and control instant food flours.

	Phytate (mg/100 g)	Tannin (mg/100 g)
*Ingredients*		
BSG	195.66 ± 5.41	178.51 ± 9.35
G. chickpea	37.62 ± 2.27	95.05 ± 4.64
Maize	129.85 ± 0.50	13.18 ± 0.00

*Blends and control*		
IF20	133.15 ± 4.93^a^	56.44 ± 4.70^a^
IF15	107.99 ± 5.13^b^	56.11 ± 4.67^a^
IF10	92.96 ± 4.97^c^	49.33 ± 4.65^a^
CSB	100.96 ± 4.35^bc^	33.24 ± 0.00^b^

IF20 (70% maize, 10% germinated chickpea, and 20% BSG), IF15 (70% maize, 15% germinated chickpea, and 15% BSG), IF10 (70% maize, 20% germinated chickpea, and 10% BSG), and CSB (corn soya blend). Values are mean ± standard deviation; the means with the same superscript letters across the column are not significantly different at *p* < 0.05. All values are taken from triplicate measurements.

**Table 5:** Sensory evaluation of the formulated instant flour porridges and the control (CSB).

Blends and control	Color	Flavour	Aroma	Taste	Mouthfeel	Overall acceptability
IF20	2.66 ± 1.00^c^	2.78 ± 0.71^b^	2.94 ± 0.84^b^	2.97 ± 0.90^b^	3.09 ± 1.09a	2.91 ± 0.86^b^
IF15	3.44 ± 1.01^b^	3.31 ± 0.78^a^	3.41 ± 0.91^b^	3.44 ± 0.98^ab^	3.47 ± 0.88a	3.50 ± 0.92^a^
IF10	3.72 ± 0.81^ab^	3.50 ± 0.95^a^	3.41 ± 0.95^b^	3.47 ± 0.98^ab^	3.38 ± 1.01a	3.53 ± 0.95^a^
CSB	4.19 ± 1.00^a^	3.65 ± 1.12^a^	3.91 ± 0.93^a^	3.69 ± 1.15^a^	3.28 ± 1.25a	3.78 ± 1.10^a^

IF20 (70% maize, 10% germinated chickpea, and 20% BSG), IF15 (70% maize, 15% germinated chickpea, and 15% BSG), IF10 (70% maize, 20% geminated chickpea, and 10% BSG), and CSB (corn soya blend). Values are mean ± standard deviation (*n* = 48); the means with the same superscript letters across the column are not significantly different at *p* < 0.05.

## Data Availability

All the supporting data are available and will be provided upon request.
